# Determinants of participation in colonoscopic screening by siblings of colorectal cancer patients in France

**DOI:** 10.1186/1471-2407-10-355

**Published:** 2010-07-06

**Authors:** Myriam Taouqi, Isabelle Ingrand, Michel Beauchant, Virginie Migeot, Pierre Ingrand

**Affiliations:** 1 Public Health; Centre Hospitalier Universitaire de Poitiers; Université de Poitiers; 6 rue de la Milétrie BP 199; 86005 Poitiers Cedex, France; 2 Epidemiology & Biostatistics, INSERM CIC-P 802; Centre Hospitalier Universitaire de Poitiers; Université de Poitiers; 6 rue de la Milétrie BP 199; 86005 Poitiers Cedex, France; 3 Hepatogastroenterology; Centre Hospitalier Universitaire de Poitiers; Université de Poitiers; 6 rue de la Milétrie BP 199; 86005 Poitiers Cedex, France

## Abstract

**Background:**

Targeted colonosocopic screening is recommended for first-degree relatives of colorectal cancer patients diagnosed before the age of 60 and offers the possibility of reducing morbidity and mortality, but participation remains too low. The objective of this study was to determine in a French population the factors that affect siblings' participation in screening, notably those relating to the individuals, their medical care, their family and their social network.

**Methods:**

A cross sectional survey was conducted in siblings of index patients having undergone surgery for colorectal cancer between 1999 and 2002 in two French counties. Siblings were contacted during 2007 and 2008 through the index patient. The factors affecting participation in colonoscopic screening were studied by logistic regression taking into account family cluster effect.

**Results:**

172 siblings of 74 index cases were included. The declared rate of undergoing at least one colonoscopy among siblings was 66%; 95%CI 59-73%. Five variables were independently associated with colonoscopic screening: perceiving fewer barriers to screening (OR = 3.2; 95%CI 1.2-8.5), having received the recommendation to undergo screening from a physician (OR = 4.9; 1.7-13.7), perceiving centres practising colonoscopy as more accessible (OR = 3.2, 1.3-7.8), having discussed screening with all siblings (OR = 3.9; 1.6-9.6) and being a member of an association (OR = 2.6; 1.0-6.6).

**Conclusions:**

The factors independently associated with participation in CRC screening by an individual at increased risk belonged to each of four dimensions relating to his individual psychosocial characteristics, to his relationship with a physician, within the family and social environment. The relevance of these results to clinical practice may help to improve compliance to recommendations in a global preventive strategy including all stages of the information pathway from the physician to the index patient and his relatives.

## Background

Approximately 25% of new colorectal cancer (CRC) cases occur in individuals who are at higher than average risk of the disease [1]. A recent meta-analysis showed that first-degree relatives (FDRs) of CRC patients, especially brothers and sisters, have twice the risk of developing the illness as compared with the general population [2]. Colonoscopic screening of FDRs of CRC patients offers the possibility of reducing morbidity and mortality [3,4]. Screening recommendations in France, advise colonoscopic screening for subjects at increased risk owing to a family history of CRC occurring in one FDR below the age of 60 or in two or more FDRs irrespective of age of onset [5]. High-risk individuals because of first-degree relatives with CRC or colorectal adenoma before the age of 60 are excluded from organized screening which concerns medium risk individuals, that is to say all subjects aged 45 or more, even if they have never had digestive disorders. As in other countries, high-risk individuals are not systematically informed about their increased CRC risk in France. Screening recommendations addressing high-risk individuals also differ from those aimed at very high risk subjects, belonging to families with a hereditary form of CRC (FAP or HNPCC). Recent data indicate that FDRs of CRC patients significantly under-use screening, the participation rate lying between 30 and 64% [6-9,11,12].

Until now, few studies on the factors associated with participation in CRC screening by FDRs, recruited subjects at increased risk as defined by the recommendations currently in force (definition of family history, age of index patient, age of FDR and type of examination recommended for this population) [13]. In these studies, relationships with a physician [6,8,9] and within the family [6,9] were consistently associated with participation in screening but little attention was paid to support from friends and colleagues [6]. Finally, the subjects who perceived the fewest barriers to screening were also the most likely to participate [6,7,9]. These four major factors that influence participation in screening by FDRs at risk of CRC - individual characteristics, recommendation from a physician, relationships within the family and the social environment - should therefore be the subject of a comprehensive study [6]. The theoretical framework used in our study was drawn up after a review of the literature about the potential relevance of validated models, corroborated by analysis of interviews carried out during a preliminary qualitative study [14]. The Health Belief Model (HBM) [15] has proved its relevance in the study of individual preventive health behaviour, especially in colorectal cancer [6-9,11] and interviews [14] brought out the four major constructs of the HBM - perceived susceptibility to and severity of CRC, perceived benefits of and barriers to participating in screening - and also referred to motivation to safeguard health [16,17]. These interviews also pointed out a normative dimension (how much the person feels social pressure to do something), which is a component of the Theory of Reasoned Action (TRA), another psychosocial model of preventive behaviour [18] which, to our knowledge, has not been studied in the context of targeted screening. A third model, the Social Network Theory (SNT) as applied to health was added to our conceptual framework to study the role of family and social environment. This model provides the structural (quantitative aspects such as number of ties and frequency of contact with ties) and functional aspects, including emotional support (receiving reassurance that one is loved and cared for) and material support (assistance provided by social network ties) [19].

To our knowledge, since the general implementation of the colorectal screening recommendations, no research has included all the key concepts derived from the three heretofore mentioned models as well as relationships within the family, the doctor-patient relationship, and factors such as perception of healthcare organization and fatalism [20,21]. The aim of this study was to identify and quantify the respective roles of these factors in determining participation in screening by the siblings of patients below the age of 60 operated on in France for CRC.

## Methods

The study's design was that of a cross sectional survey of siblings, taking into account a reasonable five-year delay between the index patients' CRC first surgery and the undertaking of colonoscopy surveillance among siblings. The index patients, retrospectively identified from hospital discharge records, responded to the following inclusion criteria: having undergone a first surgical intervention for CRC between 1999 and 2002 in a hospital in either of two counties in the west of France, namely La Vienne and Les Deux-Sèvres (population 743,416 inhabitants), being not more than 60 years old at the time of surgery, having an adequate command of French, having at least one brother or sister, and having the consent of both surgeon and patient (or surviving spouse in the case of death). Index patients were excluded in the case of hereditary cancer confirmed by genetic analysis or chronic inflammatory bowel disease. Siblings, identified through the index patients, responded to the following inclusion criteria: residing in metropolitan France, having at least one ancestor in common with the index patient, having adequate command of French, and having given signed written consent. Siblings were excluded from the study if they fell outside the criteria for screening as defined by the recommendations: personal history of CRC or chronic inflammatory bowel disease, colonoscopy performed in the context of symptoms, falling outside the age range intended by the French recommendations (less than 45 years old or being more than 5 years younger than the index patient at the time of diagnosis). The initial contact with the index patients - or their spouses - and their siblings was made by the operating surgeon, with the index patients' or surviving spouses' written consent, all in the strictest respect of medical confidentiality.

**Figure 1:**
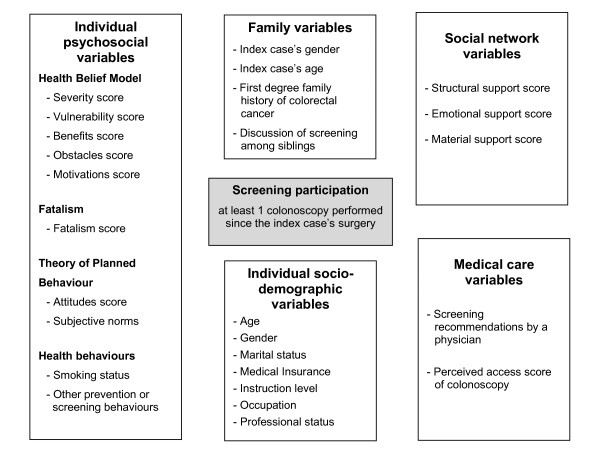
**Summary of variables and their respective dimensions included in the analysis of participation of siblings to colonoscopic screening**.

Selected variables were included in the questionnaire (Figure 1). The items from the HBM were translated from Champion's scale [16] and its subsequent revisions [17,21]. Fatalism was measured by four items adapted from Powe's index of fatalism [20]. Multi-item scales were scored by summing the responses given. The TRA was approached via its twin components of attitudes (personal) and subjective norms (social) [18]. Health-linked behaviours were also recorded [9,10]. Variables relating to the medical context included the fact of having received the recommendation to undergo screening from a physician and the perceived ease of access to centres practising colonoscopy in terms of geographic location and waiting times. Also recorded were the FDR's characteristics and family-related variables. Among social network scores, the structural support score was calculated from three items: marital status, contact with friends and family, and being a member of an association [22]. The emotional support score incorporated four items relating to the availability of friends and relatives to talk about personal or health problems; a final item dealt with material support [19]. The study received the approval of the French regulatory authorities.

The outcome was participation in screening, defined as reporting at least one colonoscopy performed since the index patient's original surgery. The data were collected between March 2007 and February 2008 by means of a self-administered questionnaire sent by post to siblings. In case of non-response, a written reminder was sent, followed by three telephone calls. Questionnaires missing responses in more than half the variables of interest on any given scale were discounted. In calculating a score, when at least half the items were answered, and when the unanswered questions were of similar content to allow an extrapolation, the average of these answers was attributed to the non-answered items.

The univariate statistical analysis compared participating with non-participating individuals using a Pearson's chi-square test or a Fisher's exact test for qualitative variables, a parametric test comparing means for quantitative variables and a Mann-Whitney non-parametric test for ordered variables, with a significance level of 5%. For quantitative variables, prior to logistic regression analysis, the hypothesis of linearity in the logit was assessed using the polynomial method and the Box-Tidwell transformation [23]. In the case of non-linearity, categorization into two classes defined by the median was carried out. The correlation between variables was computed using Spearman's correlation matrix. The variables selected at the level of P < 0.25 were entered into the initial multivariate model, with the exception of those variables for which more than 10% of the data were missing. The multivariate analysis, performed on a database with no missing data for the retained variables, followed a descending stepwise procedure, first by dimension, then on all the selected variables. Three variables were kept for adjustment: age, sex and education level. The logistic regression took into account the cluster effect resulting from the inclusion of several individuals belonging to the same family. The search for confounding factors and factors of colinearity was carried out by monitoring the variation of beta estimators and standard errors at each stage of the procedure. The search for relevant interactions was performed on the final model. The adequacy of the final model was confirmed by the Hosmer-Lemeshow test.

## Results

**Figure 2:**
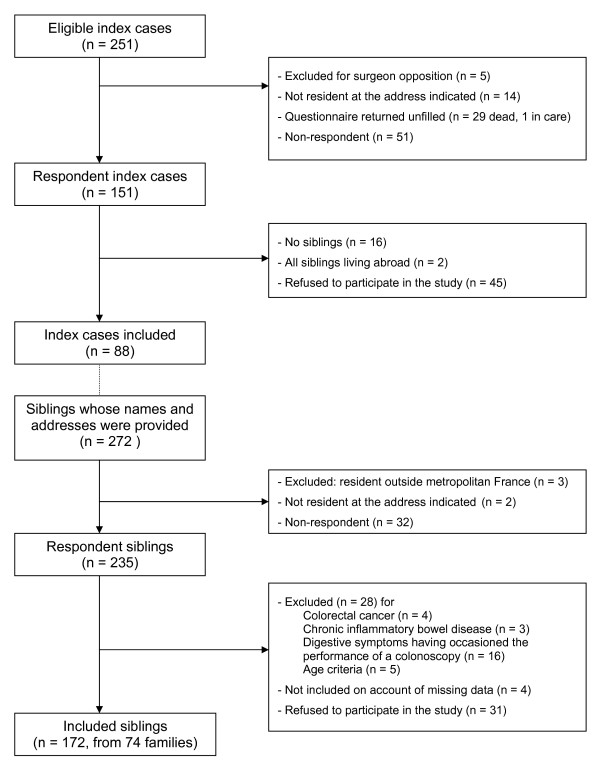
**Flow chart describing the stages in the inclusion of index patients and their siblings**.

Of the 251 index patients identified, 88 (35%) were included (Figure 2). Of the 272 brothers and sisters whose details were provided, a total of 172 (63%) were included. These were the siblings of 74 index patients and almost half of them were the latters' only brother or sister. Of the 172 brothers and sisters, 66% (95% CI 59% to 73%) reported that they participated in screening. Taking into account 31 siblings who refused to participate in the study and 32 non- respondents (although probably not all of them did meet the inclusion criteria), a low estimate of the participation rate was 45%.

Univariate analyses of associations between participation of siblings in screening and socio-demographic characteristics, individual psychosocial factors, medical factors and social network factors are presented in tables 1, 2 and 3. No socio-demographic characteristic was significantly associated with participation in screening at the 5% level. Among the individual psychosocial factors, the mean scores for perceived susceptibility to CRC (*P *= 0.0026), motivation to safeguard health (*P *= 0.025) attitudes (*P *= 0.024) and perceived benefits of screening (*P *= 0.0004) were significantly higher in subjects who participated in screening. This group perceived fewer barriers to screening (*P *< 10^-4^). These differences were observed for each of the items included in the benefits score (reassurance, lessening of anxiety, early detection of an abnormality or cancer, diminution of the risk of mutilating surgery or dying of CRC) and barriers score (embarrassment to talk about CRC and undergo regular checks, additional anxiety, unpleasant nature of the examination, time and cost implications) respectively. Fatalism seemed somewhat less marked in those who participated, but not to the extent of reaching the level of significance (*P *= 0.058). The screened brothers and sisters were more often non-smokers (*P *= 0.038), were more likely to have been told about the screening recommendations by a physician (*P *< 10^-4^), had a more favourable impression of the accessibility of colonoscopy (*P *= 0.0011), had more often discussed screening with all their brothers and sisters (*P *< 10^-4^) and had a higher mean score for emotional support (*P *= 0.039). The screened siblings were more likely to be a member of an association, but not to the extent of reaching the level of significance (*P *= 0.055).

**Table 1:** Univariate analysis of socio-demographic characteristics of index patients' siblings and their association with participation in colonoscopic screening.

	Total	Participators	Non-participators	*P*
	(n = 172)	(n = 114)	(n = 58)	
Sex: female	87 (50.6)	59 (51.8)	28 (48.3)	0.67
Age	59.8 (9.2)	59.1 (8.2)	61.2 (10.8)	0.21
Married or living with someone	135 (79.0)	89 (78.8)	46 (79.3)	0.93
Health insurance				-
Social Security with top-up	165 (95.9)	110 (96.4)	55 (94.8)	
Social Security without top-up	4 (2.3)	2 (1.8)	2 (3.5)	
Public health care	3 (1.7)	2 (1.8)	1 (1.7)	
Level of education				0.12
None or basic school-leaving certificate	80 (46.5)	50 (43.9)	30 (51.7)	
Brevet (college)	12 (7.0)	7 (6.1)	5 (8.6)	
Professional studies	53 (30.8)	35 (30.7)	18 (31.0)	
Baccalaureate	10 (5.8)	7 (6.1)	3 (5.2)	
Higher education diploma	17 (9.9)	15 (13.2)	2 (3.5)	
Profession				0.74
Farmer	15 (8.9)	10 (8.9)	5 (8.9)	
Self-employed	9 (5.4)	5 (4.5)	4 (7.1)	
Management	17 (10.1)	13 (11.6)	4 (7.1)	
Intermediate	21 (12.5)	15 (13.4)	6 (10.7)	
Employee	48 (28.6)	35 (31.2)	13 (23.2)	
Manual worker	36 (21.4)	22 (19.6)	14 (25.0)	
Retired (profession not given)	14 (8.3)	8 (7.1)	6 (10.7)	
No professional activity	8 (4.8)	4 (3.6)	4 (7.1)	
Professional status				0.85
Unemployed	6 (3.5)	5 (4.4)	1 (1.7)	
Housewife	8 (4.7)	6 (5.3)	2 (3.4)	
Retired	93 (54.4)	61 (54.0)	32 (55.2)	
Working	64 (37.4)	41 (36.3)	23 (39.7)	

Quantitative variables: mean (standard deviation). Qualitative variables: number (percentage) Missing data: marital status (1), profession (4), professional status (1).

**Table 2:** Univariate analysis of individual psychosocial factors of index patients' siblings and their association with participation in colonoscopic screening.

	Total	Participators	Non-participators	*P*
	(n = 172)	(n = 114)	(n = 58)	
Health Belief Model				
Perceived CRC vulnerability score (1 to 5)	3.5 (1.2)	3.7 (1.2)	3.1 (1.1)	0.0026
Perceived CRC severity score (2 to 10)	7.7 (2.0)	7.8 (1.9)	7.5 (2.3)	0.27
Perceived screening benefits score (6 to 30)	25.6 (4.4)	26.6 (3.6)	23.6 (5.2)	0.0004
Perceived screening barriers score (6 to 30)	14.5 (6.0)	12.9 (5.6)	17.6 (5.6)	< 10^-4^
Score for motivation to safeguard health (7 to 35)	28.0 (4.6)	28.6 (4.4)	26.9 (4.8)	0.025
Fatalism score (4 to 20)	11.1 (4.9)	10.6 (4.8)	12.1 (4.8)	0.058
Theory of Reasoned Action				
Attitude score (perceived usefulness of screening) (1 to 5)	4.2 (1.2)	4.4 (1.2)	3.9 (1.1)	0.024
Subjective norms(perceived social pressure to perform screening)	134 (88.7)	100 (98.0)	34 (69.4)	< 10^-4^
Other preventive or screening behaviours	158 (91.9)	105 (92.1)	53 (91.4)	1.0
Smoking status				0.038
Non-smoker	109 (65.7)	79 (72.5)	30 (52.6)	
Ex-smoker	36 (21.7)	19 (17.4)	17 (29.8)	
Smoker	21 (12.6)	11 (10.1)	10 (17.5)	

Quantitative variables: mean, (standard deviation). Qualitative variables: number (percentage)

Missing data: Perceived vulnerability score (4), perceived severity score (3), perceived benefits score (8), perceived barriers score (6), motivation score (6), fatalism score (4), attitude score (10), subjective norms (21), smoking status (6).

**Table 3:** Univariate analysis of factors relating to the index patients' siblings' relationships with their physicians, families and friends and the association of these factors with participation in colonoscopic screening.

	Total	Participators	Non-participators	*P*
	(n = 172)	(n = 114)	(n = 58)	
Medical care				
Advice by a physician to undergo screening	65 (40.4)	58 (53.7)	7 (13.2)	< 10^-4^
Score for perceived accessibility of colonoscopy centres (1 to 5)	3.8 (1.2)	4.0 (1.2)	3.3 (1.1)	0.0011
Family				
Index patient sex: female	84 (48.8)	58 (50.9)	26 (44.8)	0.45
Index patient age at time of diagnosis				0.49
≤ 45 years old	22 (12.8)	16 (14.0)	6 (10.3)	
> 45 years old	150 (87.2)	98 (86.0)	52 (89.7)	
Number of cases of colorectal cancer declared in first-degree relatives				0.1
0	45 (26.2)	26 (22.8)	19 (32.8)	
1	116 (67.4)	79 (69.3)	37 (63.8)	
≥ 2 *	11 (6.4)	9 (7.9)	2 (3.4)	
Discussion about screening with all brothers and sisters	84 (49.7)	73 (65.2)	11 (19.3)	< 10^-4^
Social network				
Belonging to a social group	54 (32.0)	41 (36.9)	13 (22.4)	0.055
Number of contacts with friends or relatives per month				0.24
≤ 2	19 (12.6)	13 (12.6)	6 (12.5)	
3 to 11	98 (64.9)	63 (61.2)	35 (72.9)	
≥ 12	34 (22.5)	27 (26.2)	7 (14.6)	
Structural support score (0 to 4)	2.3 (1.1)	2.4 (1.1)	2.1 (0.9)	0.13
Emotional support score (0 to 4)	3.4 (1.0)	3.5 (0.9)	3.2 (1.2)	0.039
Material support	153 (91.6)	103 (93.6)	50 (87.7)	0.24

Quantitative variables: mean, (standard deviation). Qualitative variables: number (percentage)

Missing data: advice by a doctor to undergo screening (11), perceived accessibility score (12), discussion of screening with all brothers and sisters (3), belonging to a social group (3), number of contacts with friends and relatives (21), structural support score (24), emotional support score (1), material support (5).

* 1 individual reported 4 cases of colorectal cancer in FDRs not documented by genetic analysis.

The factors favouring siblings' participation in screening derived from the multivariate logistic regression are shown in table 4. The subjects who perceived less barriers to screening (OR = 3.2), had received the recommendations for screening from a physician (OR = 4.9), perceived colonoscopy as more accessible (OR = 3.2), had discussed screening with all their brothers and sisters (OR = 3.9) and were members of an association (OR = 2.6) were significantly more likely to participate in screening.

**Table 4:** Factors favouring siblings' participation in colonoscopic screening according to multivariate logistic regression with cluster effect (n = 138).

	Adjusted OR	95% CI	P
Less perceived barriers (score < 14)	3.2	[1.2 - 8.5]	0.022
Screening advised by a physician	4.9	[1.7 - 13.7]	0.0025
Centres practising colonoscopy perceived as more accessible (score ≥ 4 )	3.2	[1.3 - 7.8]	0.011
Screening discussed with all brothers and sisters	3.9	[1.6 - 9.6]	0.0037
Belonging to a social group	2.6	[1.0 - 6.6]	0.044
Age (< 60 ans/≥ 60 ans)	1.7	[0.7 - 4.4]	0.26
Sex (female/male)	1.3	[0.6 - 3.0]	0.50
Level of education (≥ Baccalaureate/< Baccalaureate)	0.9	[0.3 - 2.7]	0.86

Three variables were kept for adjustment in the final model: age, sex and education level. Intra-familial correlation coefficient of the residuals = 0.14.

## Discussion

This study showed that the factors independently associated with participation in CRC screening by an individual at increased risk belonged to each of four dimensions relating to his individual psychosocial characteristics and his relationship with a physician, within the family and social environment. Univariate analysis confirmed that screening participants were more likely to be non-smokers [10], a factor linked to health behaviour. Their scores for perceived susceptibility to CRC [7], perceived benefits of screening and motivation to safeguard health were higher, whilst their scores for perceived barriers to screening [6,7,9] and fatalism were lower. Their perception of the usefulness of screening and in particular their sensitivity to social pressure was higher. More often they had received recommendations from their physicians [6,8,9] and their score for perceived accessibility of colonoscopy centres was higher. A significant number of them had discussed screening with their brothers and sisters [6,9] and were members of an association. Their score for emotional support was higher. Factors that had not previously been studied in the context of targeted CRC screening were identified (fatalism, accessibility of centres practising colonoscopy, social pressure, being a member of an association and support of a friend or colleague).

The study population was defined as subjects at increased risk of CRC on account of their family history, as defined by the current recommendations. The CRC patient's age and the histological diagnosis of the tumour were taken from the medical record, thus permitting an accurate definition of subjects at increased risk of CRC; this is in contrast to several studies relying on self-reporting of family history [13]. FDRs who had undergone colonoscopy on account of symptoms or in the context of surveillance relating to their own medical history were excluded from the analysis in order to avoid artificially increasing the rate of participation in screening. Despite this, we found a participation rate of 66%, which is relatively high compared with data in the literature [6-12]. However, the results must be interpreted in the light of the inherent limits of a declarative study. We suspected two sources of selection bias in our study. First, index patients' acceptance to let us contact their relatives might have introduced a bias towards an overrepresentation of families with good interrelationship (which has been identified as a determinant of participation in colonoscopic screening). Second, we may postulate that those relatives who declined participation in the study might likely have a lower concern with screening. Thus results may be biased directed towards an overestimated adherence.

The passing on of information by a physician was the factor most strongly associated with participation. This result has been found in several studies, be it targeted [6,8,9] or generalized mass screening, and it is potentially at this level that interventions designed to increase screening participation should be implemented [24,25]. In the context of targeted screening, the relationships within the family have a very specific impact and are equally predictive of the behaviour of FDRs [6,9]. Discussion about screening amongst siblings involves the index patient divulging his illness and being educated to play the role of passing on medical information about the increased risk and about screening, two aspects that are difficult to address [12,25] since as for other hereditary pathologies (familial hypercholesterolaemia [26], breast cancer, melanoma ...), physicians are not authorized to contact their patients' FDRs directly. In order to prevent under-diagnosis in their relatives, index patients, assuming that they themselves have been adequately informed of the risks by their physicians, must first of all pass on this information to their FDRs. Then these latter must feel themselves to be personally at risk and decide to enrol in a screening programme.

Factors of the HBM, in particular the barriers, the perceived benefits and susceptibility, with the exception of perceived severity, were strongly related to screening behaviour of siblings. These results are consistent with a review of the literature [15] in which, if perceived susceptibility was the most important dimension associated with adopting a preventive behaviour, perceived barriers were the dimension that best explained behaviour, while the influence of perceived severity was very limited. In the context of screening, this may result from the absence of symptoms and of personal experience of the illness concerned. Perceived benefits play a greater role in behaviour when one is already ill than in prevention. Perceived barriers to colonoscopy included the unpleasant nature of the examination, its cost, increased anxiety, the embarrassment of talking about CRC or having to undergo regular screening [27,28] and the time it takes. These results suggest that measures aimed at increasing participation should seek to lower each of the perceived barriers.

The study equally demonstrated the role played by ease of access to colonoscopy in terms of distance to travel and waiting times for targeted screening. Thus, the barriers to undergoing a colonoscopy include cognitive and emotional factors, logistical barriers and barriers relating to the organization of treatment services (geographic location of screening centres and waiting times for appointments).

The study enabled us to take into account the role of the social network in the context of targeted screening, certainly the most difficult area to interpret. It is an important variable in determining behaviour; people with a more extensive social network and more frequent contact with other people are more likely to adopt preventive behaviour (cancer screening) and more likely to engage in health promotion activities (balanced diet, tobacco avoidance) [19], but up until now this variable has rarely been studied in the context of targeted screening for CRC [6], and that before the implementation of the screening recommendations. In this study, the influence of the global structural support provided by the social network could not be satisfactorily interpreted because of the significant amount of missing data. However, the role played by being a member of an association, demonstrated in the United States in individuals at medium risk [19], was also found. Moreover, emotional support emerged as a relevant factor. The part played by social support, and especially family support in this case, should be evaluated in subsequent studies.

Randomized trials have been conducted on FDRs of CRC patients using tailored interventions aimed at Health Belief Model factors, in particular perceived barriers to screening. The results suggest that an improvement in screening participation could be obtained from tailored interventions rather than by simply making information about colorectal cancer generally available [29,30].

## Conclusions

Participation of siblings of CRC patients in the recommended screening requires further efforts to be improved. Identification of the multiple factors associated with participation in targeted screening underscores the interest of a global approach including all the stages in the information pathway from the physician, to the index patient and his relatives. Improving the effectiveness of preventive strategies depends on increasing physicians' awareness of the importance of identifying individuals at increased risk and of delivering recommendations about screening, on educating patients and helping them to pass on information about their illness to their relatives and on taking into account the various individual psychosocial factors.

## Competing interests

The authors declare that they have no competing interests.

## Authors' contributions

MT contributed to, performed statistical analysis and prepared the manuscript. II contributed to study concept and design, data acquisition and control, statistical analysis and manuscript edition. MB and VM contributed to study concept and manuscript edition. PI contributed to study concept and design, statistical analysis and manuscript edition, and supervised the study. All authors read and approved the final manuscript.

## Pre-publication history

The pre-publication history for this paper can be accessed here:

http://www.biomedcentral.com/1471-2407/10/355/prepub
